# Biochemical aspects of nitric oxide synthase feedback regulation by nitric oxide

**DOI:** 10.2478/v10102-011-0012-z

**Published:** 2011-06

**Authors:** Jana Kopincová, Angelika Púzserová, Iveta Bernátová

**Affiliations:** 1 Department of Physiology, Jessenius Faculty of Medicine, Comenius University, Martin, Slovak Republic; 2 Institute of Normal and Pathological Physiology, Slovak Academy of Sciences, Bratislava, Slovak Republic

**Keywords:** nitric oxide synthase, feedback regulation, inflammation, NF-κB

## Abstract

Nitric oxide (NO) is a small gas molecule derived from at least three isoforms of the enzyme termed nitric oxide synthase (NOS). More than 15 years ago, the question of feedback regulation of NOS activity and expression by its own product was raised. Since then, a number of trials have verified the existence of negative feedback loop both in vitro and in vivo. NO, whether released from exogenous donors or applied in authentic NO solution, is able to inhibit NOS activity and also intervenes in NOS expression processes by its effect on transcriptional nuclear factor NF-κB. The existence of negative feedback regulation of NOS may provide a powerful tool for experimental and clinical use, especially in inflammation, when massive NOS expression may be detrimental.

## Physiological function of nitric oxide synthase feedback regulation

Nitric oxide (NO) is a small gas molecule participating in physiological processes in diverse cells from protozoan parasite *Leishmania donovani* to mammalian neurocytes (Basu *et al*., 1997). Nevertheless, many biochemical characteristics of its synthesis remain as yet unknown.

It seems almost unbelievable what a long time has passed since Hermann found in 1865 that NO combines with hemoglobin. Later NO was shown to react with the heme groups and nearly hundred years later the kinetics and equilibrium of the reaction of NO with hemoglobin was described (Gibson & Roughton, 1957). The ability of NO to activate heme protein guanylate cyclase and to increase the level of cyclic guanosine monophosphate (cGMP) in various tissues (Arnold *et al*., 1977) raised the question about the physiological role of NO and the ensuing quest for an answer gave birth to the discovery of NO-mediated cGMP-dependent vasorelaxation (Rapoport & Murad, 1983).

During the following ten years, the nature of divergent physiological functions of NO was discovered along with distinct isoforms of NO-synthesizing enzyme.

## Nitric oxide synthase

The enzyme, which NO is derived from, bears the name ‘nitric oxide synthase’ (NOS, EC 1.14.13.39). To date, NOS has been purified in at least three isoforms, which can be distinguished by their origin from different genes, diverse localization within the cell, specific regulation and various sensitivity to inhibitors, with about 51–57% homology between the human isoforms (Geller & Billiar, 1998; Alderton *et al*., 2001).

The typical nomenclature of NOS isoforms is derived from the tissue of the first isolation, although occurrence of particular isoforms is not strictly limited to a certain type of cells. Thus the isoform first purified from rat brain tissue is called **neuronal NOS** (nNOS) or NOS I (Bredt *et al*., 1990). In addition to neurons, nNOS may be exprimed also in skeletal muscles, lung epithelial cells, kidneys, adrenal glands, skin, hypophysis, vascular smooth muscle cells and other cells and tissues (Boulanger *et al*., 1998; Förstermann *et al*., 1998; Esper *et al*., 2006).

NO synthesized by nNOS participates primarily in neurotransmission and neuromodulation. In the nucleus tractus solitarii and rostral ventrolateral medulla, the function of nNOS is related to central control of blood pressure (Chang *et al*., 2003; Lin *et al*., 2007). In the periphery, NO acts as neurotransmitter in perivascular vasodilatory nerves named ‘nitrergic’ and sometimes is considered to be the main neurotransmitter of the inhibitory non-adrenergic non-cholinergic system (Antošová *et al*., 2005). However, in the case of eNOS knock-out mice, nNOS was also able to supply the role of eNOS in vasorelaxation (Meng *et al*., 1998). Additionally, nNOS-derived NO functions also in synaptic plasticity, including hippocampal long-term potentiation, and plays an important role in stress and adaptive responses of the organism (Bon & Garthwaite, 2003; Púzserová *et al*., 2006; Bernátová *et al*., 2007a).

**Endothelial NOS** (eNOS) or NOS III was purified from bovine aortic endothelial cells (Pollock *et al*., 1991) and may be found also in cardiomyocytes, hepatocytes, thrombocytes, vascular smooth muscle cells, lung epithelial cells and others (Förstermann *et al*., 1998; Arnal *et al*., 1999; Strapková *et al*., 2008). In the cell, eNOS is typically targeted into plasmalemmal invaginations termed ‘caveolae’, which inhibits eNOS function. Increase in intracellular Ca^2+^ concentration, *e.g.* by shear stress, leads to the formation of calcium/calmodulin complex (Ca^2+^/CaM), which enables eNOS to dissociate from caveolae and become catalytically active (Alderton *et al*., 2001). Endothelial NO has a variety of physiological functions, including vasodilatation, inhibition of thrombocyte adhesion and aggregation and antiatherogenic effects (Esper *et al*., 2006).

Both isoforms mentioned above are considered to be expressed constitutively. At least in some tissues their activity is Ca^2+^-dependent and their NO production reaches picomolar levels (Strapková & Nosálová, 2001).

**Inducible NOS** (iNOS) or NOS II is the last of three isoforms of NO-synthesizing enzyme, purified for the first time from activated macrophages (Hevel *et al*., 1991). By contrast to so-called constitutive isoforms (nNOS and eNOS), iNOS had been earlier thought to be Ca^2+^-independent and expressed after induction under inflammatory conditions (Geller & Billiar, 1998).

While induction of iNOS expression in murine and rat cells requires incubation with just one bacterial lipopolysacharide (LPS), IL-1β, IL-6, TNF-α or an other compound, in the majority of human cells it requires a complex cytokine combination (Kleinert *et al*., 2004). After stimulation, a variety of cells are able to express iNOS, *e.g.* hepatocytes, monocytes, mast cells, cardiac myocytes, glial cells or vascular smooth muscle cells (Michel & Feron, 1997; Aktan, 2004). In contradistinction to the generally held opinion, some specific cells such as murine ileal epithelium, epithelium of bronchi and bronchioles of lamb and sheep or human airway epithelium showed also constitutive expression of iNOS (Guo *et al*., 1995; Hoffman *et al*., 1997; Sherman *et al*., 1999). This may be further enhanced in the presence of certain factors such as LPS (Gath *et al*., 1996) or oxidative stress (Cooke & Davidge, 2002) and, on the contrary, suppressed by glucocorticoid treatment (Guo *et al*., 1995).

Once induced, iNOS produces continuously high levels of NO up to micromolar range, until the enzyme is degraded (Geller & Billiar, 1998). The high output of NO from iNOS acts in antimicrobial, antiviral, antiparasitic and tumoricidal processes (MacMicking *et al*., 1997; Geller & Billiar, 1998) and the cytotoxic effect of NO is involved in immunological and tissue-damaging actions (Bogdan, 2001). On the other hand, excessive production of NO participates in the pathophysiology of several autoimmune diseases (for example Crohn's disease, rheumatoid arthritis), chronic inflammatory diseases (such as asthma), acute lung injury and meconium aspiration syndrome or various degenerative diseases (Kröncke *et al*., 1998; Bogdan, 2001).

## Enzymatic action of NOS

Only a homodimeric enzyme is able to produce NO coupling L-arginine oxidation to NADPH consumption and releasing L-citrulline as coproduct. Several cofactors are necessary for stable dimerization of NOS and NO synthesis. For each monomer, heme and flavins (flavin adenine dinucleotide and flavin mononucleotide) as prosthetic groups are required to tightly bind to the molecule. Ca^2+^/CaM and 5,6,7,8-tetrahydrobiopterin (BH_4_) binding to monomer provide both dimerization and solely enzymatic action, while zinc ion is required one per dimer for stabilization (Geller & Billiar, 1998; Alderton *et al*., 2001).

Ca^2+^/CaM binding to NOS dimer reflects intracellular Ca^2+^ concentration and if it decreases, the bond dissociates and electron transport stops (Daff *et al*., 1999). This is the basis for the activity of constitutively expressed NO synthases to be calcium-regulated. Synthesis of NO during catalytic activity of iNOS had been formerly considered to be Ca^2+^-independent, but contrary to previous views, the activity of iNOS isolated from guinea-pig lungs could be inhibited by chelation of Ca^2+^ ions (Shirato *et al*., 1998). Likewise, human iNOS seems to require at least a low level of calcium for optimal binding of calmodulin (Geller & Billiar, 1998).

Yet another factor, BH_4_, is crucial for physiological action of NOS, especially for iNOS (Alderton *et al*., 2001). In the absence of BH_4_ (or L-arginine), the phenomenon called “uncoupling” occurs, which means that NADPH consumption proceeds independently of L-arginine oxidation. NOS will utilize NADPH, but in that case the product of reaction is superoxide anion (O_2_^−^) and H_2_O_2_ (Gorren & Mayer, 2002). At limiting concentrations, the situation becomes more complicated. If there is just one BH_4_ per NOS dimer, the BH_4_-free subunit will produce O_2_^−^ in the uncoupled reaction, while the BH_4_-supplemented subunit will produce NO (Gorren *et al*., 1996). Taking into consideration that NO and superoxide can react together rapidly forming peroxynitrite, NO synthase may act as peroxynitrite synthase (Andrew & Mayer, 1999). As a consequence, the bioavailability of BH_4_ for NOS further declines, as peroxynitrite oxidizes BH_4_ to inactive dihydro-L-biopterin (Milstien & Katusic, 1999) and more NOS reactions become uncoupled.

Excessive formation of O_2_^−^ or peroxynitrite after cytokine-mediated NOS expression, for example in acute lung injury (such as in meconium aspiration syndrome), may exceed the capacity of the oxidant defense system leading to oxidative stress. This may potentiate the lung injury and inhibit lung surfactant production (Mokra & Mokry, 2007; 2010)

## Feedback regulation of NOS activity

Potential NO-mediated oxidative damage requires detailed regulation of NOS activity as well as NOS expression.

Besides carefully managed conditions of dimer activation by cofactors, the question of feedback regulation of NOS activity and expression by its own product has been raised when Rogers & Ignarro (1992) found that during *in vitro* determination of NOS activity the rate of L-citrulline formation was not linear. The above mentioned authors showed that addition of oxyhemoglobin (a strong NO scavenger) made the rate of NO formation linear, while addition of superoxide dismutase (which increases the half-life of NO) inhibited NOS activity and made the rate of NO production more non-linear. As the decrease of NOS activity was observed even after admixing authentic NO or exogenous NO donors to the enzymatic reaction, the authors for the first time hypothesized the existence of binding between heme-iron and NO, which was considered to represent a negative feedback regulation of NOS activity (Rogers & Ignarro, 1992).

Consequently, the assay was repeated with iNOS from activated murine macrophages (Assreuy *et al*., 1993) and rat alveolar macrophages (Griscavage *et al*., 1993), which means that feedback regulation is not only a matter of "constitutive" isoforms. Since then, a number of trials verified the existence of this negative feedback loop both in vitro and in vivo (Park *et al*., 1994; Buga *et al*., 1993; Ravichandran *et al*., 1995; Grumbach *et al*., 2005; Bernátová *et al*., 2007b; Zhen *et al*., 2008).

### Chemical basis of feedback regulation of NOS activity

The chemical basis of NO-NOS interaction is not completely understood. The heme-iron bond in the NOS molecule may occur in both reduced (ferrous) and oxidized (ferric) form and even formation of both ferric- and ferrous-nitrosyl complexes with NOS exists (Hurshman & Marletta, 1995), leading to NOS inhibition.

The ferrous-nitrosyl complex is considered to be a natural part of catalysis (Abu-Soud *et al*., 1995) and it is formed during the first seconds after NO synthesis initiation by the heme binding of newly generated NO (Santolini *et al*., 2001b). Particular NOS isoforms differ by the rate of autoinhibition. For example, the majority (70–90%) of nNOS was present at its ferrous-nitrosyl complex regardless of the NO concentration in solution (Abu-Soud *et al*., 1995). By contrast, iNOS heme-NO complex consists of a rather ferric-nitrosyl complex formed rapidly and depending on NO concentration, although a minor amount of ferrous heme-NO complex forms in iNOS, suggesting that its regulation also involves generated NO binding (Santolini *et al*., 2001b). Regarding eNOS, it seems to form relatively little heme-NO complex with the lowest formation rate (Abu-Soud *et al*., 2000).

For all iNOS, eNOS and nNOS, a loss of activity appears when heme binds NO that accumulates in a solution as a consequence of chemical equilibrium (Santolini *et al*., 2001a). To examine the susceptibility of the particular NOS isoforms to self-inhibition by NO, Scott and his colleagues (2002) constructed inhibition curves of each NOS isoform to NO donor S-nitroso-N-acetyl-penicillamine (SNAP). The calculated IC_50_ values for SNAP were 1 800 µM for iNOS, 200 µM for eNOS and 51 µM for nNOS. The high level of NO produced by inducible isoform may thus inhibit the activity of constitutive isoforms, which becomes especially apparent under conditions of increased iNOS activity, *e.g.* in sepsis (Scott *et al*., 2002) or meconium-induced inflammation (Li *et al*., 2001).

The existence of negative feedback regulation may contribute to the beneficial effect of inhaled NO on persistent pulmonary hypertension or meconium aspiration syndrome in newborns (Ichinose *et al*., 2004). However, to date data about the direct effect of inhaled NO on pulmonary inflammation processes are missing.

## Feedback regulation of NOS expression

After the feedback regulation of NOS activity by NO had been proven, the next step was to determine whether feedback regulation of NOS expression existed and the attention of scientists focused on the regulatory action of both exogenous and endogenous NO.

The first observation of NO intervention in transcriptional processes was made by Park *et al*. (1994) who incubated the culture of astroglial cells with hemoglobin and found increased iNOS mRNA after induction compared to the control, which was completely abolished in the presence of exogenous NO donor.

### Chemical basis of feedback regulation of NOS expression

Now it is clear that NO, whether released from exogenous donors or applied in authentic NO solution, is able to inhibit iNOS expression in concentrations close to the physiological range (Colasanti *et al*., 1995). This is true also for eNOS. The presence of NO donor reduced the rate of eNOS mRNA increase, which is the physiological reaction of endothelial cells to laminar shear stress (Grumbach *et al*., 2005).

We also know that the promoter region of iNOS gene contains several binding sites for NF-κB (Hecker *et al*., 1997), which plays a central role in the regulation of NOS expression (Colasanti *et al*., 1995; Kleinert *et al*., 2004). This clarifies also the linkage between inflammation and iNOS expression. The inhibitory effect of NO on NF-κB was proved also for eNOS (Grumbach *et al*., 2005, Figure 1), however, there is no accordance about the site of NO inhibitory action on NF-κB. According to what has been found, NO may inhibit activation of NF-κB (Colasanti *et al*., 1995), NF-κB binding to DNA (Park *et al*., 1997) or induce and stabilize the inhibitor of NF-κB (Peng *et al*., 1995; Davis *et al*., 2004). In addition, it is possible that NO intervenes with feedback regulation of NOS expression at multiple levels. Further, feedback regulation of NOS expression was assumed to be transmitted by cGMP as a decrease of eNOS expression was found after pretreatment with exogenous cGMP (Vaziri & Wang, 1999). From this point of view, the molecular tracks of a negative loop between NO and NOS isoform expression has not been satisfactorily elucidated as yet.

**Figure 1:**
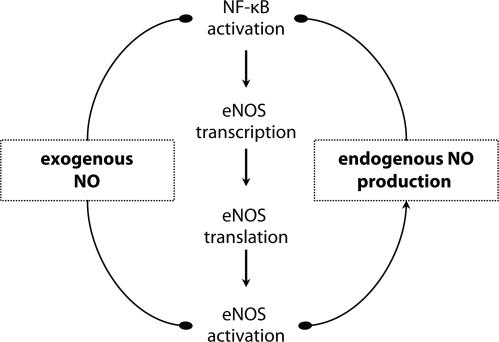
Negative feedback regulatory effect (——•) of nitric oxide (NO) to endothelial nitric oxide synthase (eNOS) expression mediated via nuclear factor κB (NF-κB).

## Oxidative stress and NOS expression

Considering NF-κB contribution to NOS expression, we cannot neglect the role of oxidative stress. The production of reactive oxygen species (ROS) during exercise led to NF-κB activation and, afterwards, to expression of both eNOS and iNOS in rat skeletal muscles (Gomez-Cabrera *et al*., 2005). Moreover, induction of oxidative stress by glutathione depletion caused up-regulation of renal and aortic eNOS and iNOS in animals (Zhen *et al*., 2008). This finding is logical, because under conditions of oxidative stress, the NO regulatory process of NOS expression may be interrupted. As O_2_^−^ serves as NO scavenger (forming peroxynitrite), the bioavailability of NO for the tissue is limited. The following up-regulation of NOS has to compensate NO deficiency. However, under conditions of oxidative stress, the essential cofactors of NO synthesis may be inactivated and NOS itself may produce ROS and thus worsen the situation. Participation of ROS in compensatory NOS expression became apparent after antioxidant treatment where eNOS and iNOS were down-regulated in rat kidneys, aorta and heart (Vaziri *et al*., 2000).

## Aims for the future

The existence of negative feedback regulation of NOS expression and activity by its product NO provides a powerful tool for experimental and clinical use. Chronic administration of low doses of NOS inhibitor enhances NOS activity and NO production in vascular tissues via feedback regulation (Kopincová *et al*., 2008). Thus NO, whether inhaled or derived from exogenous NO donors, should stop processes leading to massive iNOS expression and activity in situations when it is detrimental, *e.g.* in inflammatory processes. However, the ”user's manual” for this tool needs to be further elucidated.
